# Severe Manifestations of Chikungunya Fever in Children, India, 2016

**DOI:** 10.3201/eid2409.180330

**Published:** 2018-09

**Authors:** Pradeep K. Sharma, Maneesh Kumar, Girraj K. Aggarwal, Virender Kumar, R.D. Srivastava, Ashish Sahani, Rohit Goyal

**Affiliations:** Sri Balaji Action Medical Institute, New Delhi, India

**Keywords:** sepsis, septic shock, chikungunya fever, children, viruses, India, chikungunya virus

## Abstract

Chikungunya is a relatively benign disease, and a paucity of literature on severe manifestations in children exits. We describe a cohort of pediatric chikungunya fever patients in New Delhi, India, who had severe sepsis and septic shock, which can develop during the acute phase of illness.

Chikungunya fever, regarded as a benign disease with infrequent severe manifestations, has caused epidemics in many countries in the past decade. The worst epidemic of chikungunya fever in Delhi, India, occurred in 2016 (*1*). Severe manifestations of chikungunya fever in adults have been reported recently (*2*–*7*); however, there is a paucity of similar data in children. The objective of our study was to describe the characteristics of pediatric patients who had atypical or severe forms of the disease and to search for predictive factors for severe forms.

## The Study

We conducted a retrospective, observational study in the pediatric intensive care unit (PICU) and pediatric high-dependency unit of a tertiary care hospital in New Delhi, India. We included patients whose chikungunya infection was diagnosed by positive real-time reverse transcription PCR (RT-PCR) during September–December 2016. The RT-PCR was done using a Gene Finder DENV/CHKV Real*Amp* Kit (Osang Healthcare, Gyeonggi-do, South Korea) at Oncquest Laboratories (New Delhi, India). This qualitative assay uses a 1-tube RT-PCR technique with internal control for amplification and detection of chikungunya virus RNA. The study protocol was approved by the hospital’s Institutional Research Council. 

The information recorded consisted of demographic features, clinical features, laboratory parameters, course, organ dysfunction, ventilation days, inotropic days, hospital stay, and whether the patient died. We classified the disease as severe in the presence of severe sepsis, septic shock, or organ dysfunction, which were defined according to standard definitions (*8*).

A total of 49 children had chikungunya fever; 36 had nonsevere disease and 13 had severe disease. All patients with severe disease were admitted to the PICU; 11 had illness consistent with the case definition of severe sepsis and septic shock, and 2 had acute liver failure. Of the 36 patients with nonsevere disease, 16 were admitted to the PICU (11 had seizures, 4 had fluid-responsive shock, 1 had peripheral cyanosis and mottling) and 20 were admitted to the pediatric high-dependency unit (3 had bleeding manifestations, 4 had severe abdominal pain, 2 had underlying cyanotic congenital heart disease, 2 had body temperature >40.3°C with irrelevant talking, 7 had dehydration, and 2 had severe rash). The median age was 12 years for patients with severe disease and 6.5 years for patients with nonsevere disease; male sex predominanted in both groups (Table). Frequency of fever, body ache, arthralgia, and vomiting were similar for both groups. Peripheral cyanosis, along with mottling of skin and encephalopathy, was significantly higher in the group with severe disease. Serum albumin was significantly lower in the group with severe disease (3 vs. 3.75 g/dL).

**Table:** Demographic, clinical, and laboratory features at admission of pediatric patients with severe and nonsevere chikungunya fever, New Delhi, India, 2016*

Parameters	Nonsevere disease, n = 36	Severe disease, n = 13	p value†
Median age, y (range)	6.5 (0.75–15)	12 (0.5–14)	0.28
Male:female ratio	3.5:1	2.25:1	0.53
Age group			
Infant, 1 mo–1 y	3 (8.3)	4 (30.7)	0.06
Toddler, 2–5 y	13 (36)	1 (7.7)	
School age, 6–12 y	3 (8.3)	0	
Adolescent, 13 to <18 y	17 (47.2)	8 (61.5)	
Clinical profile			
Fever	36 (100)	13 (100)	
Body ache	10 (27.7)	4 (30.7)	0.83
Rash	15 (41.6)	8 (61.5)	0.22
Arthralgia	5 (13.9)	3 (23)	0.44
Vomiting	15 (41.6)	5 (38.4)	0.84
Seizures	11 (30.5)	1 (7.7)	0.14
Bleeding	3 (8.3)	4 (30.7)	0.16
Abdominal pain	4 (11)	5 (38.4)	0.25
Peripheral cyanosis and mottling of skin	2 (5.5)	10 (76.9)	**0.00**
Encephalopathy	0	3 (23)	**0.01**
Laboratory test results, median (range)			
Hemoglobin, g/dL	12.2 (6.6–17.5)	11.6 (8–13.5)	0.08
White cell count/µL	8,195 (3,700–15,200)	11,200 (4,100–44,800)	0.058
Platelet count, × 10^3^/µL)	203 (25–362)	192 (13–362)	0.61
AST, IU/L	44 (22–174)	43 (16–8,837)	0.96
ALT, IU/L	20 (9–96)	24 (8–2,311)	0.26
Albumin	3.75 (3.5–4)	3.3 (1.6–3.5)	**0.006**
Urea, mg/dL	21 (11–55)	36 (13–87)	0.094
Creatinine, mg/dL	0.4 (0.3–1.2)	0.6 (0.2–1.3)	0.37
APTT, s, control 28.4 s	NA	41.7 (27–247)	
PT, s, control 13.3 s	NA	22.3 (18.5–117)	
International normalized ratio	NA	1.77 (1.3–12.4)	
Organ dysfunction			
Cardiovascular	0	11 (84.6)	
Respiratory	0	3 (23)	
Hematological	0	5 (38.4)	
Neurologic	0	3 (23)	
Renal	0	2 (15.3)	
Hepatic	0	3 (23)	
Course and outcome			
Mechanical ventilation	0	3 (23)	
Inotropic support	0	11 (84.6)	
Renal replacement	0	2 (15.3)	
Hospital stay, d (range)	3 (2–7)	5 (2–13)	**0.0015**
Death	0	1 (7.7)	

*Values are no. (%) patients except as indicated. ALT, alaninine aminotransferase; APTT, activated partial thromboplastin time; AST, aspartate aminotransferase; NA, not applicable; PT, prothrombin time. †Categorical variables were compared using the χ^2^ test or Fisher exact test, as appropriate, and continuous variables were compared by using the nonparametric Mann-Whitney test. p values <0.05 were considered statistically significant and are shown in bold type.

Of the 11 children with septic shock, 8 were admitted to the hospital within 24 hours of developing fever; 9 had hypotensive shock, and 2 had compensated shock. In this group, 6 children required 1 vasoactive agent, 3 children required 2 vasoactive agents, and 2 children required 3 vasoactive agents. Dopamine was used in 8 patients, dobutamine in 5 patients, epinephrine in 2 patients, and norepinephrine in 2 patients. The median duration of vasoactive support was 56 hours (range 31–114 hours), and the median vasoactive inotropic score in the first 24 hours was 10 (range 5–90; score >15–20 is considered serious). A vasoactive inotropic score >20 was seen in 2 children. Mean pH was 7.26 (reference range 7.35–7.45), mean lactate 5.1 mmol/L (reference range <2 mmol/L), mixed venous saturation 55% (reference range 70%–80%), and mean base excess at admission −7.7 mEq (reference range –2 to 2 mEq). Of the 2 children with acute liver failure with encephalopathy, 1 had dengue virus (positive dengue IgM by enzyme immunoassay) and the other had hepatitis E virus (reactive anti–hepatitis E IgM by enzyme immunoassay) co-infection.

The usual symptoms of chikungunya are fever, rash, and joint pain. Children can have features distinct from adults, such as more frequent dermatological and hemorrhagic manifestations and less frequent rheumatologic manifestations (*7*). Most patients with symptomatic disease have mild to moderate illness. A recent pediatric study reported severe disease in infants and neonates; however, septic shock was not well defined (*9*). Recently, sepsis and septic shock in adults have been described in the literature, with relatively high death rates (36%–100%) (*2*–*6*). 

## Conclusions

Our study reports a cohort of pediatric chikungunya fever patients who had severe sepsis and septic shock. In our study, children <1 year of age and 11–14 years of age were more likely to have septic shock. Most of these children were admitted to the hospital within 24 hours of developing fever, with peripheral cyanosis and cold extremities. Although children can have cold extremities during high fever, in our cohort central capillary refill time was also prolonged, and generalized skin mottling was present. In addition, these children had hypotension or metabolic evidence of poor perfusion (high lactate and low mixed venous saturation). Children with early shock had generalized erythema and diffuse edema. 

Although dopamine was the most used inotropic agent overall, in infants dobutamine was more helpful in improving shock, both clinically and metabolically. Of the infants, 3 of 4 required only dobutamine; 1 required dopamine as well. 

Shock in children who were admitted early usually resolved around the time of mitigation of fever. All 3 children who were admitted late had multiorgan failure and required mechanical ventilation. Of these, 2 had myocardial dysfunction and required multiple inotropic agents and renal replacement therapy. Both of these children had severe ascites; the child who died had bilateral pleural effusion, pericardial effusion, generalized confluent ecchymosis, and pregangrenous changes at peripheral sites. 

Our cohort of septic shock patients did not reveal clinical or microbiological evidence of other infections. Results of tests for dengue nonstructural protein 1 antigen, dengue IgM and IgG (by enzyme immunoassay), leptospira IgM and IgG (by immuno-chromatographic assay), Weil Felix serology (by latex agglutination), blood cultures, and other relevant cultures were all negative.

Although chikungunya usually has a mild course, severe life-threatening manifestations can occur. Clinicians should be aware that these manifestations can develop within 24 hours of the onset of illness, and a high index of suspicion is required to establish diagnosis. In our study, age <1 year and 11–14 years were predictive of severe disease. Further studies are required to clarify the clinical spectrum and risk factors associated with severe disease.
